# Maxillo Ostio Choanal Polyp

**DOI:** 10.4274/balkanmedj.2017.0656

**Published:** 2018-01-20

**Authors:** Süha Ertuğrul, Serdar Ensari

**Affiliations:** 1 Department of Otorhinolaryngology, Karabük University School of Medicine, Karabük, Turkey

Solitary polyps that originate in the paranasal sinuses or adjacent structures and extend to the nasopharynx are called choanal polyps. Depending on the sinus where they originate, they are termed antrochoanal, sphenochoanal, frontochoanal or ethmoidochoanal polyps. This report presents the case of an ostio-choanal polyp originating in the maxillary sinus ostium mucosa. It was termed a maxillo-ostio-choanal polyp.

A 49-year-old female patient presented to our clinic with symptoms of two-sided nasal congestion, the feeling of a foreign body in the throat and difficulty in swallowing, which had been present for 1 year. In her endoscopic nasal examination, a polypoid structure, of which the pedicle originated in the lateral nasal wall on the left, extended to the nasopharynx and obstructed the choana on both sides (Figure 1a, 1b). In her paranasal sinus tomography scan, both maxillary sinuses were observed to have a soft-tissue density consistent with a retention cyst and choanal polyp extending from the choana to the nasopharynx (Figure 1c, 1d). The patient was diagnosed with choanal polyp and operated on under general anaesthesia. Uncinectomy was performed with a trans-nasal endoscopic approach. It was observed that the choanal polyp was originating directly in the maxillary sinus ostium mucosa (Figure 1e). A cystic structure was seen inside the maxillary sinus; however, its connection with the choanal polyp could not be identified. The maxillary sinus ostium was extended, and the polyp was excised with its pedicle. The excised polyp was trans-orally removed. The polyp size was measured at 2.3x2.0 cm (Figure 1f). The cystic lesion was excised with the aid of angled telescopes and punches. The result of the histopathological examination was reported as inflammatory polyp. Written informed consent was obtained from the patient.

Some studies reported that the antrochoanal polyps most often passed through the natural ostium (2) while other studies reported that it more often passed through the accessory ostium (3), thereby extending into the nasal cavity. However, no cases of choanal polyp originating directly in the maxillary sinus ostium mucosa have been reported previously. It was deemed appropriate to designate polyps originating directly in the ostia mucosa as “ostio-choanal polyp” by affixing the term with the name of the sinus where it originated. Our case was described as a maxillo-ostio-choanal polyp.

In conclusion, choanal polyps may also develop in the ostia mucosa of sinuses. The term maxillo-ostio-choanal polyp was deemed appropriate in cases where it originates in the maxillary sinus ostium mucosa.

## Figures and Tables

**Figure 1:**
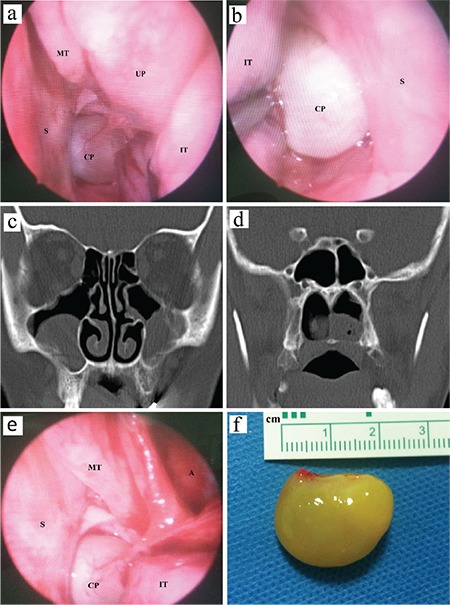
Endoscopic view of the left nasal cavity (a) and the right nasal cavity (b), Coronal CT view of the sinuses demonstrating bilateral maxillary opacification (c) and soft-tissue opacification in the nasopharynx (d), During endoscopic surgery, view of the choanal polyp originating from maxillary sinus ostium mucosa (e), Macroscopic appearance of the solitary polyp (f). *A: antrum; CP: vhoanal polyp; IT: inferior turbinate; MT: middle turbinate; S: septum; UP: uncinate process*

## References

[1] Min YG, Chung JW, Shin JS, Chi JG (1995). Histologic structure of antrochoanal polyps. Balkan Medical Journal.

[2] Kamel R (1990). Endoscopic transnasal surgery in antrochoanal polyp. Balkan Medical Journal.

[3] Hardy G (1957). The chonal polyp. Balkan Medical Journal.

